# Isolation, Pathogenicity and Genomic Analysis of *Mannheimia haemolytica* Strain XJCJMh1 in Bovine-Mycoplasma Co-Infection

**DOI:** 10.3390/microorganisms13102258

**Published:** 2025-09-26

**Authors:** Chengzhe Liang, Kashaf Kareem, Lichun Zhang, Yafei Liang, Huiying Wu, Beibei Li, Jinliang Sheng

**Affiliations:** 1 College of Animal Science and Technology, Shihezi University, Shihezi 832003, China; lcz261@outlook.com (C.L.); kashafkareem55@stu.shzu.edu.cn (K.K.); zlc1226@outlook.com (L.Z.); 13899390597@163.com (Y.L.); why18889949487@outlook.com (H.W.); 2 Shawan Municipal Bureau of Agriculture and Rural Affairs, Shawan 832100, China; 17599935665@163.com

**Keywords:** *Mannheimia haemolytica*, *Mycoplasma bovis*, whole genome sequencing, pathogenicity

## Abstract

Mixed infections of *Mannheimia haemolytica* and *Mycoplasma bovis* are relatively common in bovine respiratory diseases, presenting severe respiratory symptoms and high mortality that severely endanger the cattle industry. In this study, a serotype A1 strain of *Mannheimia haemolytica*, designated as XJCJMh1, was isolated and identified from the lung tissue of a hybrid Simmental calf infected with *Mycoplasma bovis*. The pathogenicity of this strain was evaluated using Kunming mice as a model. The results indicated that infection with XJCJMh1 caused pathological manifestations such as pulmonary hemorrhage and edema in mice. Subsequently, the genome of this strain was sequenced and assembled using Illumina sequencing to obtain general genomic features. The genome was annotated and analyzed for gene functions using the Swiss-Prot, NR, GO, COG, KEGG, CAZy, TCDB, and Pfam databases. Additionally, the virulence factors and resistance genes of this strain were annotated using the PHI, VFDB, and CARD databases. The genome of *Mannheimia haemolytica* XJCJMh1 is 2,595,489 base pairs (bp) in length, with a GC content of 40.93%. Notably, this strain exhibits three distinct genomic islands and contains 98 effectors associated with the type III secretion system (T3SS). The XJCJMh1 strain harbors 74 virulence genes and 45 resistance genes. We annotated the proteins, genes, and associated GO and KEGG pathways of the XJCJMh1 strain; exploring the relationship between these annotations and the strain’s pathogenicity is of considerable value. This study is of great significance for clarifying the pathogenic mechanism and genetic characteristics of the *Mannheimia haemolytica* strain XJCJMh1 in cattle, and its results provide a scientific reference for analyzing the genomic basis of pathogenicity and drug resistance of *Mannheimia haemolytica* under co-infection conditions.

## 1. Introduction

Against the backdrop of the growing global demand for beef and dairy products, the cattle industry—one of the core pillars of global animal husbandry—sustains the agricultural economies of many countries with its high output value. However, bovine respiratory diseases, which cause direct annual economic losses exceeding billions of dollars, represent a leading constraint on the sustainable development of the industry. Bovine Respiratory Disease Complex (BRDC), also known as Shipping Fever Pneumonia, is a multifactorial respiratory disease in cattle and stands as one of the most prevalent ailments in the cattle industry, frequently inflicting significant economic losses [1]. The viral pathogens associated with BRD primarily include bovine herpesvirus type 1 (BHV-1), parainfluenza 3 virus (PI3), bovine viral diarrhea virus (BVDV), bovine coronavirus (BCoV), and bovine respiratory syncytial virus (BRSV). The primary bacterial pathogens are *Mannheimia haemolytica*, *Pasteurella multocida*, *Histophilus somni*, and *Mycoplasma bovis* [2]. Studies have shown that *Mannheimia haemolytica* is the pathogen most closely linked to acute BRD [3]. Traditionally, *Mycoplasma bovis* has been regarded as a pathogen associated with chronic pneumonia in cattle. However, recent research indicates that *Mycoplasma bovis* can also induce acute BRD [4,5]. This may be attributed to the interaction between *Mycoplasma bovis* and *Mannheimia haemolytica*, which could render it another contributing pathogen for BRD [6].

Multiple studies from different regions have reported interactions between *Mycoplasma bovis* and *Mannheimia haemolytica* in bovine respiratory disease (BRD): Barrington et al. [3] found in a U.S. feedlot study that the presence of *M. bovis* in nasal swabs at 7 days post-arrival significantly increased the prevalence of *Mannheimia haemolytica* by 45% by day 28, suggesting that M. bovis may facilitate the secondary colonization of *Mannheimia haemolytica*. D’Angelo et al. [7] noted in Italian dairy farm research that M. bovis was detected in 16.16% of fatal calf pneumonia cases, with 16.62% of these cases co-infected with *Mannheimia haemolytica*, indicating their co-occurrence in severe pathological conditions. Lombardo et al. [8] observed in a cross-border study on beef steers transported from France to Italy that the co-infection rate of *Mannheimia haemolytica* and *M. bovis* increased from 16% at departure to 82.8% post-arrival, highlighting that transport stress exacerbates their co-occurrence. Howerth et al. [9] identified in Canadian pneumonic lung sample analysis that *Mannheimia haemolytica* is a primary pathogen in severe fibrinous bronchopneumonia, a condition often involving co-infections with pathogens like *M. bovis*—collectively, these findings demonstrate that the interaction between the two pathogens is a global phenomenon in BRD, influenced by management practices and environmental factors, though the current study does not provide additional data on this specific interplay.

*Mannheimia haemolytica* (MH), formerly *Pasteurella haemolytica*, is a Gram-negative, facultative anaerobic coccobacillus belonging to the Pasteurellaceae family [10]. As an opportunistic pathogen, it typically colonizes the nasal cavity and tonsillar crypts of the upper respiratory tract in ruminants [11] and can induce severe diseases (e.g., bovine pneumonia, ovine contagious pleuropneumonia) when hosts are immunocompromised or under environmental stress [12]. Based on the capsular A antigen, MH is classified into 12 serotypes (A1, A2, A5, A6, A7, A8, A9, A12, A13, A14, A16, A17), with serotypes A3, A4, A10, A11, A15 reclassified into other species [13]. In cattle, A1 and A6 are the most pathogenic, while A2 is less virulent; serotype A1 is a primary, highly pathogenic agent of the Bovine Respiratory Disease Complex (BRD) [14]. MH pathogenicity depends on synergistic virulence factors (leukotoxin, LPS, capsular polysaccharide, outer membrane proteins, and iron acquisition systems) that enable immune evasion, tissue colonization, and host proliferation [15]. However, the molecular mechanisms of *Mannheimia haemolytica*-*Mycoplasma bovis* co-infections—including virulence factor synergy and the role of antibiotic resistance genes in disease progression—remain unclear. Additionally, whether MH genomic traits (e.g., pathogenicity islands, secretion systems) enhance virulence during mixed infections has not been systematically explored. Resolving these gaps is critical to understanding how co-infections drive severe bovine respiratory disease and high mortality.

Thus, in this study, we isolated and purified a *Mannheimia haemolytica* strain from lung tissues of a *Mycoplasma bovis-infected* Simmental crossbred calf in Changji, Xinjiang. Capsular serotyping confirmed it as serotype A1. We then performed mouse pathogenicity assays and high-throughput genome sequencing, yielding genomic and functional insights to support future studies of its virulence, antibiotic resistance, and public health relevance.

## 2. Materials and Methods

### 2.1. Experimental Materials

The sample was collected from a 6-month-old Simmental crossbred calf that died on a farm in Changji, Xinjiang Uygur Autonomous R. To assess the pathogenicity of *Mannheimia haemolytica* strain XJCJMh1, twelve 40-day-old healthy female Kunming mice (purchased from Beijing Vital River Laboratory Animal Technology Co., Ltd., Beijing, China) were used. During the experiment, all animals were individually housed in designated cages at the animal facility of Shihezi University under controlled and monitored conditions. The animal experiments were conducted in accordance with ethical standards and approved by the Animal Experiment Ethics Committee (Approval No. A2024-646), which ensures that all procedures comply with ethical guidelines and prioritize animal welfare in scientific research region, China. Ethical consent was obtained from the cattle owner in compliance with guidelines for the care and use of research animals, and the animal-based research was approved by the owner.

### 2.2. Mycoplasma Bovis Detection

Soybean-sized pieces of lesioned lung and spleen tissues were separately placed into 5 mL EP tubes containing 2 mL of sterile PBS. After being minced, magnetic beads were added to each tube, and the mixtures were homogenized at 30 f/s for 10 min using a high-throughput homogenizer. Subsequently, the homogenates were centrifuged at 1000 rpm for 30 s, and the supernatants were filtered through 0.45 μm filter membranes to remove impurities. A 500 μL aliquot of each filtrate was inoculated into *Mycoplasma bovis*-specific culture medium for cultivation, while another 1 mL aliquot of each filtrate was used for genomic DNA extraction with a DNA extraction kit. Meanwhile, specific primers targeting the *uvrC* gene of *Mycoplasma bovis* were designed (sequences see Table 1), and after synthesis, PCR detection was performed using the extracted genomic DNA as templates.

### 2.3. Isolation and Identification of Mannheimia haemolytica

Bovine lung tissues collected under sterile conditions were cut into 1.0 cm × 1.0 cm × 0.5 cm pieces and placed into EP tubes containing magnetic beads. The tissue was homogenized in a high-throughput homogenizer at 30 Hz for 10 min. The EP tube was centrifuged at 1000 rpm for 30 s, and the supernatant was inoculated into Trypticase Soy Broth (TSB; purchased from Qingdao Haibo Biotechnology Co., Ltd., Qingdao, China) liquid medium. The culture was incubated at 37 °C in a shaking incubator at 200 r/min for 12–16 h to enrich the bacterial population. The enriched culture was streaked onto Tryptose Soya Agar (TSA; purchased from Qingdao Haibo Biotechnology Co., Ltd.) solid medium and incubated at 37 °C for 12–16 h. Single colonies were selected and re-cultured in TSB liquid medium at 37 °C with shaking at 200 r/min for 12 h. The bacterial suspension containing the isolated strain was sent to Youkang Biotechnology Co., Ltd. (Xinjiang, China) for sequencing. The obtained sequences were trimmed to remove low-quality ends and subjected to BLAST (Software’s version 2.16.0) analysis against the NCBI database. The BLAST results were compared with reference sequences of target species to confirm the isolate’s identity. Specific primers for *Mannheimia haemolytica* (targeting the *gcp* gene) were designed based on sequence alignments [16]. Bacterial DNA was extracted using the TIANamp Bacterial DNA Kit, and polymerase chain reaction (PCR) was performed for species identification. The extracted DNA served as the template for PCR detection, with primer sequences listed in Table 1.

### 2.4. Serotyping of Mannheimia haemolytica

The serotyping of the bacterial strain was conducted using the PCR-based method established by Klima et al. [17] for identifying the capsular serotypes 1 (*Hyp*), 2 (*Core2*), and 6 (*TupA*) of *Mannheimia haemolytica*. The primer sequences used for the PCR assays are detailed in Table 1.

### 2.5. Animal Pathogenicity Experiment

The isolated and purified *Mannheimia haemolytica* was revived and cultured to the logarithmic growth phase. The bacterial suspension was diluted, and its concentration was adjusted to 0.5 McFarland units (1.5 × 10^8^ cfu/mL) using a McFarland nephelometer. Twelve 40-day-old Kunming white mice were randomly divided into an experimental group and a control group, with six mice in each group. Each mouse in the experimental group was intraperitoneally injected with 0.2 mL of bacterial suspension, while each mouse in the control group was injected with 0.2 mL of PBS. During the experiment, the feeding conditions, mental status, and mortality rate of the mice were systematically monitored and recorded at regular intervals. Dead mice were dissected to observe pathological changes in the heart, lungs, spleen, liver, and kidneys. At 24 h, surviving mice were anesthetized with isoflurane, followed by cardiac blood collection and euthanasia via cervical dislocation. Lung tissue exhibiting gross lesions—including congestion, hemorrhage, consolidation, and frothy exudate on the pleural surface—was aseptically isolated for bacterial identification.

### 2.6. Histopathological Examination

The lungs, spleens, and livers from the deceased cattle and mice were collected and fixed in 10% formalin for 72 h. The tissues were then processed via alcohol dehydration and embedded in paraffin wax. Using standard histopathological techniques, paraffin sections were prepared and stained with hematoxylin and eosin (H&E). The stained sections were examined under a light microscope to observe histopathological changes.

### 2.7. Library Construction and Genome Sequencing, Assembly

The whole-genome sequencing was performed by Novogene (Beijing) Co., Ltd., Beijing, China. Libraries were prepared using standard Illumina workflows, including end repair, A-tailing, adapter ligation, PCR amplification, and size selection, followed by final evaluation and analysis.

### 2.8. Genomic Component Analysis and Functional Annotation

GeneMarkS software (version 4.17) was employed for the prediction and filtering of coding genes [18]. For the identification of interspersed and tandem repeats, RepeatMasker (version 4.0.5) [19] and TRF (Tandem Repeats Finder, version 4.07b) [20] were utilized, respectively. The prediction of non-coding RNAs (ncRNAs) was conducted as follows: tRNAs were predicted using tRNAscan-SE [21], which identifies tRNA regions and their secondary structures. rRNAs were detected by aligning sequences to the rRNA database (with a default identity threshold of ≥50%) and using rRNAmmer [22] to predict novel and unannotated rRNAs. The results from both approaches were combined. sRNAs were first annotated by comparing against the Rfam database and then confirmed using the cmsearch program (version 1.1rc4). Genomic islands, prophages, and CRISPR arrays were predicted using IslandPath-DIOMB [23], phiSpy (Nov11_v2.3) [24], and CRISPRdigger [25], respectively.

Functional annotation was performed by aligning the predicted genes against the GO, KEGG, COG/KOG, NR, Pfam, CAZy, TCDB, and Swiss-Prot databases using BLAST (Software’s version 2.16.0) (blastp, evalue ≤ 1 × 10^−5^). Annotations were selected based on the highest score with identity ≥ 40% and coverage ≥ 40%. Signal peptides and transmembrane structures were predicted using SignalP (version 4.1) and TMHMM (version 2.0c), respectively, to identify secretory proteins. T3SS effectors were predicted using effectorP (version 1.0.1), and secondary metabolite gene clusters were identified using antiSMASH (version 4.0.2). Virulence and antibiotic resistance genes were predicted by aligning against the PHI, VFDB, ARDB, and CARD databases.5.

## 3. Results

### 3.1. Detection Results of Mycoplasma bovis

**Figure 1:**
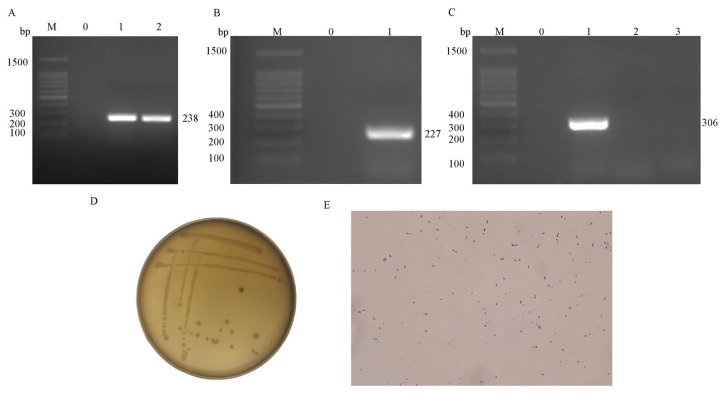
PCR Detection of *Mycoplasma bovis* and Identification and Morphological Characteristics of *Mannheimia haemolytica*. Details are as follows: (**A**) shows the PCR amplification results of *Mycoplasma bovis*, where M represents a 100 bp DNA ladder, 0 is the blank control, and 1 and 2 are the PCR results of bovine lung tissue sample and bovine spleen tissue sample, respectively; (**B**) shows the PCR amplification results of *Mannheimia haemolytica*, where M is a 100 bp DNA ladder, 0 is the blank control, and 1 is the amplification product using specific primers for the gcp gene of this bacterium; (**C**) shows the PCR amplification results of *Mannheimia haemolytica* serotypes, where M is a 100 bp DNA ladder, 0 is the blank control, and 1, 2, and 3 are the amplification products using specific primers for Hyp, Core2, and TupA genes, respectively; (**D**) shows the colony morphology of *Mannheimia haemolytica* cultured on TSA solid medium (observed by naked eye); (**E**) shows the cell morphology of this bacterium after Gram staining of its bacterial solution (observed under a 100× oil immersion objective).

PCR results of genomic DNA extracted from lesioned lung and spleen tissues of the cattle indicated that the animal was infected with *Mycoplasma* (Figure 1A).

### 3.2. Isolation, Identification, and Microscopic Examination of Mannheimia haemolytica

The results of agarose gel electrophoresis show that a bright band of 227 bp was amplified using the specific primer gcp for *Mansheimia hemolytic* (Figure 1B), which is consistent with the expected size of *Mansheimia hemolytic*, indicating that the strain is *Mansheimia hemolytic*, and it is named XJCJMh1.

The results of agarose gel electrophoresis show that the strain has a clear band at 306 bp between 300 bp and 400 bp (Figure 1C), which confirms that the serotype of strain XJCJMh1 is type A1.

### 3.3. Postmortem Examination of Infected Cattle

**Figure 2:**
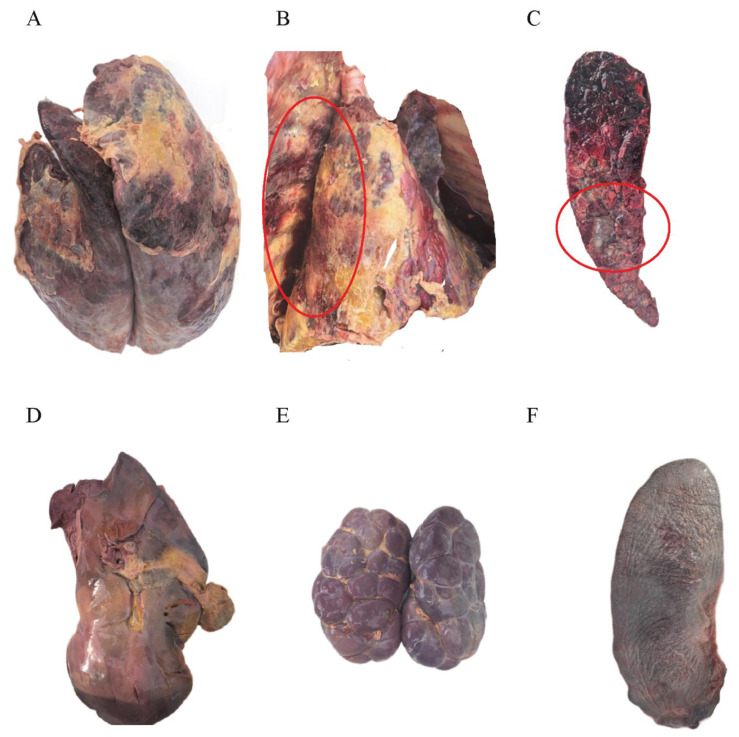
Postmortem Images of Diseased Cattle. (**A**). Lung tissue morphology showing typical fibrinous pneumonia. (**B**). Adhesions between lung tissue and the pleural cavity. (**C**). Cross-section of the lung revealing extensive caseous necrosis. (**D**). Liver tissue morphology. (**E**). Kidney. (**F**). Spleen.

The lungs of the deceased cattle exhibited typical fibrinous pneumonia, with severe congestion and necrosis of the lung tissue (Figure 2A) and adhesions (Figure 2B). Upon sectioning the lungs, a large amount of caseous necrosis was observed (Figure 2C). The liver showed severe congestion and edema, appearing dark red (congested) with some areas being soft in texture (necrotic) (Figure 2D). The spleen and kidneys were markedly enlarged, congested, and necrotic (Figure 2E,F).

### 3.4. Mouse Lethality Assay

**Figure 3:**
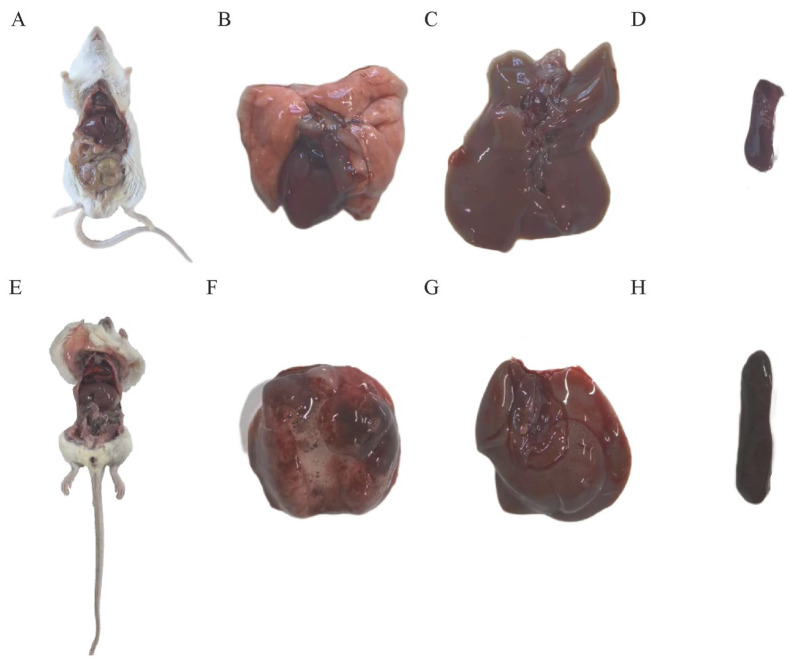
Histopathological Examination of Mice in the Pathogenicity Experiment. (**A**). Gross anatomy of control mice. (**B**). Normal lung morphology of control mice. (**C**). Normal liver morphology of control mice. (**D**). Normal spleen morphology of control mice. (**E**). Gross anatomy of experimental mice. (**F**). Lung morphology of experimental mice, showing congestion, edema, and pleural adhesions. (**G**). Liver morphology of experimental mice, showing congestion and edema. (**H**). Spleen morphology of experimental mice, showing congestion and edema.

To investigate the virulence of the *Mannheimia haemolytica* strain XJCJMh1, we conducted an infection experiment in mice using an inoculum dose of 1.5 × 10^8^ cfu/mL. The results showed significant differences in physiological responses between the experimental and control groups 4 h post-inoculation. Mice in the control group exhibited no abnormal symptoms, whereas those in the experimental group began to show signs of depression, anorexia, reduced activity, huddling, and somnolence 4 h after infection with *Mannheimia haemolytica*. Some mice also experienced tremors. After 6 h, rapid breathing and mortality were observed in the experimental group. We immediately performed autopsies on the deceased mice and found varying degrees of pathological changes in the organs of the infected mice. Specifically, the lungs of control mice appeared normal (Figure 3B), while those of the infected mice showed congestion, edema, and pleural adhesions (Figure 3E,F). Additionally, the spleens and livers of the infected mice were mildly enlarged (Figure 3G,H). Bacterial liquid PCR identification confirmed the infection in all mice, as indicated by consistent electrophoresis band sizes (Figure S1).

### 3.5. Observation of Pathological Tissue Sections

**Figure 4:**
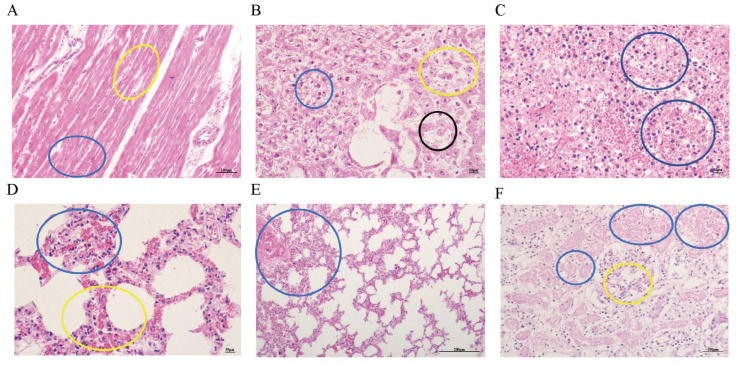
Histopathological Sections of Organs from Diseased Cattle. (**A**). Histopathological section of bovine heart tissue (200× magnification). (**B**). Histopathological section of bovine liver tissue (400× magnification). (**C**). Histopathological section of bovine spleen tissue (400× magnification). (**D**). Histopathological section of bovine lung tissue (400× magnification). (**E**). Histopathological section of bovine lung tissue (100× magnification). (**F**). Histopathological section of bovine kidney tissue (200× magnification).

Histopathological examination revealed edema widening between myocardial fibers in the diseased cattle (as indicated by the blue circle in Figure 4A); capillary congestion between myocardial fibers was also observed (as indicated by the yellow circle in Figure 4A). The liver exhibited a large number of inflammatory cells, with widened spaces between hepatic cords, loose arrangement of hepatocytes, and atrophy (as indicated by the yellow circle in Figure 4B); hepatocellular steatosis was present (as indicated by the blue circle in Figure 4B), and some hepatocyte nuclei showed signs of dissolution and necrosis (as indicated by the black circle in Figure 4B). The spleen showed a disorganized structure with a large number of red blood cells and inflammatory cells, and partial necrosis was observed (as indicated by the blue circle in Figure 4C). The lungs had a large number of inflammatory cells and widened interstitial spaces (as indicated by the yellow circle in Figure 4D); severe congestion and dilation of the microcirculation in the pulmonary arterioles and alveolar walls were evident (as indicated by the blue circle in Figure 4D,E). Renal interstitial hemorrhage was observed, with a large number of necrotic renal tubules (as indicated by the blue circle in Figure 4F); glomerular capillaries showed severe congestion (as indicated by the yellow circle in Figure 4F).

**Figure 5:**
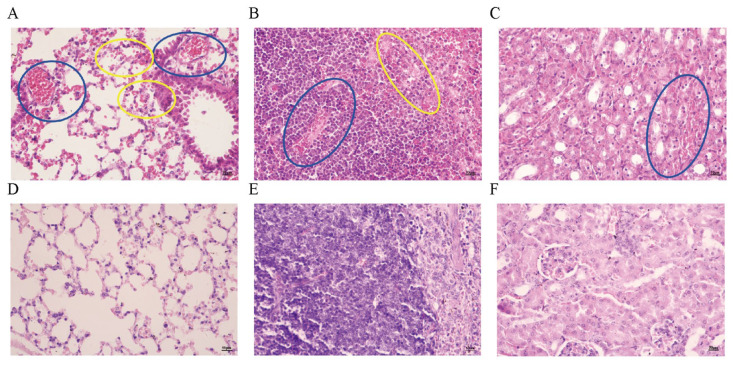
Histopathological sections of tissues from infected mice. (**A**). Histopathological section of the lung from infected mice (400×). (**B**). Histopathological section of the spleen from infected mice (400×). (**C**). Histopathological section of the kidney from infected mice (400×). (**D**). Histopathological section of the lung from normal mice (400×). (**E**). Histopathological section of the spleen from normal mice (400×). (**F**). Histopathological section of the kidney from normal mice (400×).

Severe congestion in the lungs of infected mice is visible (as indicated by the blue circle in Figure 5A), along with a small amount of hemorrhage (as indicated by the yellow circle in Figure 5A). The spleen shows severe hemorrhage (as indicated by the blue circle in Figure 5B) and a small amount of cellular necrosis (as indicated by the yellow circle in Figure 5B). The renal interstitium exhibits severe hemorrhage (as indicated by the blue circle in Figure 5C).

### 3.6. Genome Assembly and Whole-Genome Component Analysis Results

**Figure 6:**
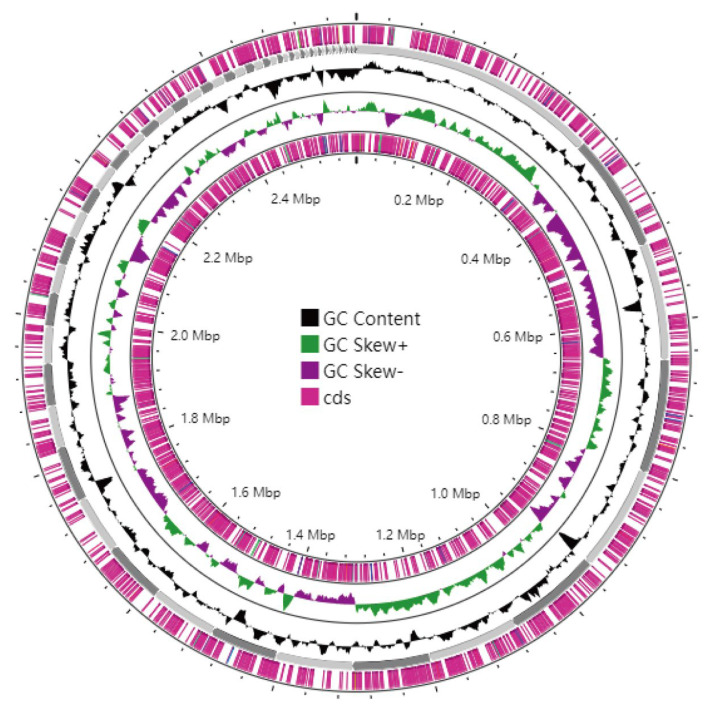
Whole-genome circle map of strain XJCJMh1, which was generated using the CGView website B. Genomic Island Structure of Strain XJCJMh1.

The genome of strain XJCJMh1 was assembled de novo, and the scaffold information has been deposited in GenBank under accession number SUB15374783. The assembly consists of 58 scaffolds with a total length of 2,595,489 bp (2.60 Mb). The longest scaffold is 336,678 bp, and the shortest is 1953 bp. The N50 is 104,215 bp, and the N90 is 23,753 bp. The G + C content of the genome is 40.93%. A total of 2644 coding genes were predicted, with coding regions accounting for 88.22% of the total genome length. The visualization of the genome assembly is shown in Figure 6. In addition to the coding genes, the genome contains 157 tandem repeat sequences, 362 dispersed repeat sequences, 82 non-coding RNAs, 3 genomic islands, 20 prophages, and 20 CRISPR sequences. The overall results of the genome components are summarized in Table 2

**Figure 7:**
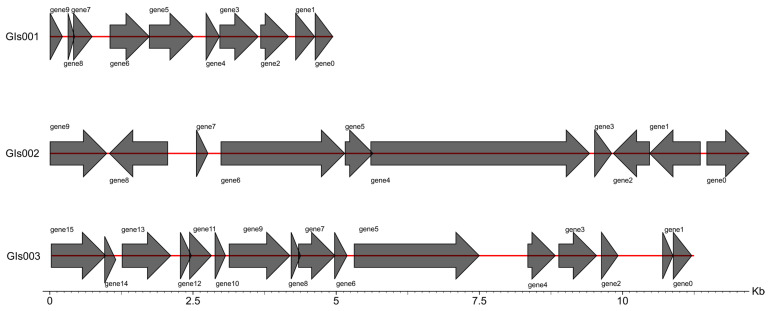
Genomic Island Structure of Strain XJCJMh1. Arrows indicate the direction of the sequence. A right arrow represents the sense strand, and a left arrow represents the antisense strand. When the number of genes on the positive strand is greater than that on the antisense strand, the color of the middle line is red; otherwise, it is black. (The functional annotations of the genes in the genomic islands are provided in the Supplementary Table S1).

Genomic islands are segments of DNA that have been integrated into the microbial genome through horizontal gene transfer, often originating from bacteria, phages, or plasmids. These islands can be associated with various biological functions, including pathogenic mechanisms and host adaptation. In the genome of strain XJCJMh1, three genomic islands were predicted: Gls001, Gls002, and Gls003. The total length of these islands is 28,042 bp, as shown in Figure 7.

### 3.7. Gene Function Annotation and Prediction Results

**Figure 8:**
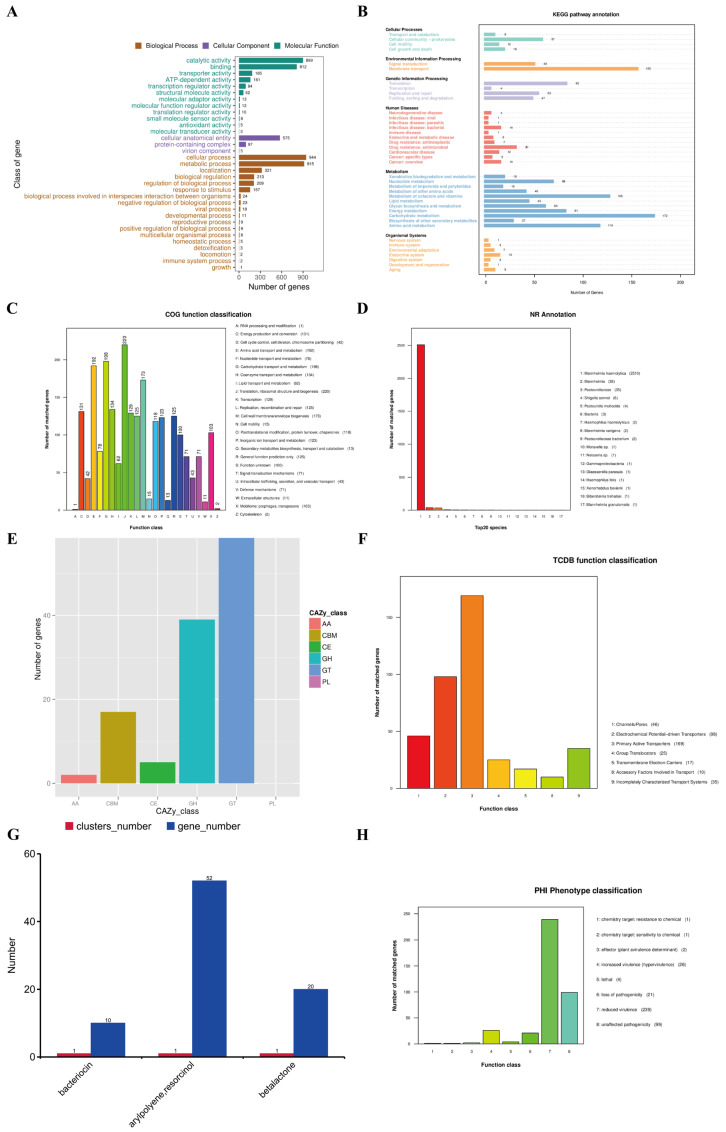
(**A**). GO Functional Classification of the Genome of Strain XJCJMh1. (**B**). KEGG Metabolic Pathway Classification of the Genome of Strain XJCJMh1. (**C**). COG Functional Classification of the Genome of Strain XJCJMh1. (**D**). NR Functional Classification of the Genome of Strain XJCJMh1. (**E**). CAZy Functional Classification and Gene Count Statistics of the Genome of Strain XJCJMh1. (**F**). TCDB Functional Classification of the Genome of Strain XJCJMh1. (**G**). Secondary Metabolite Gene Clusters and Gene Count Statistics of the Genome of Strain XJCJMh1. (**H**). PHI Annotation Results Statistics of the Genome of Strain XJCJMh1.

The genome of strain XJCJMh1 was annotated for gene functions using the GO, KEGG, COG, NR, CAZy, and TCDB functional databases. The Gene Ontology (GO) annotations were categorized into three major classes: Cellular Component, Molecular Function, and Biological Process. The Cellular Component category includes cellular anatomical entities, protein complexes, and viral components. The Molecular Function category primarily consists of catalytic activity and binding, as well as transport and transcriptional regulatory activities. The Biological Process category is dominated by cellular and metabolic processes, followed by localization, biological regulation, and regulation of biological processes. It also includes immune system processes and biological adhesion. These annotations provide a comprehensive overview of the functional roles of the genes within the genome of strain XJCJMh1, as illustrated in Figure 8A.

The KEGG PATHWAY annotation classification result (Figure 8B) indicates that the annotated genes of strain XJCJMh1′s genome are mainly distributed across six major categories and 39 pathways, including Cellular Processes, Environmental Information Processing, Genetic Information Processing, Human Diseases, Metabolism, and Organismal Systems. Among these, the category with the highest number of annotated genes is Metabolism, particularly in key pathways related to bacterial growth and reproduction, such as carbohydrate metabolism, amino acid metabolism, and cofactor and vitamin metabolism.

The COG annotation classification result (Figure 8C) shows that the most abundant gene categories in the genome of strain XJCJMh1 are J (Translation, ribosomal structure and biogenesis), E (Amino acid transport and metabolism), and G (Carbohydrate transport and metabolism). This observation is consistent with the results of KEGG analysis, highlighting the importance of these pathways in bacterial life processes.

The NR (Non-Redundant Protein Database) is a comprehensive protein sequence database that integrates sequences from multiple sources such as GenBank, PDB, Swiss-Prot, PIR, and PRF. The annotation result (Figure 8D) of the genome of strain XJCJMh1 using the NR database shows that the most annotated genes are from the hemolytic Mansheimia itself. This finding indicates that the isolated strain is indeed hemolytic Mansheimia.

The CAZy (Carbohydrate-Active enZYmes) database is a comprehensive resource that categorizes enzymes involved in the degradation, modification, and biosynthesis of carbohydrates into five main classes: Glycoside Hydrolases (GHs), GlycosylTransferases (GTs), Polysaccharide Lyases (PLs), Carbohydrate Esterases (CEs), and Auxiliary Activities (AAs). In the genome of strain XJCJMh1, a total of 127 genes were annotated using the CAZy database. Among these, the most abundant category was GlycosylTransferases (GTs), with 64 annotated genes. This was followed by Carbohydrate Esterases (CEs), which had 39 annotated genes. Notably, no genes were annotated in the Polysaccharide Lyases (PLs) category. These findings highlight the significant roles of GTs and CEs in the metabolic processes of strain XJCJMh1, particularly in carbohydrate metabolism and modification. The results are presented in Figure 8E.

The TCDB (Transporter Classification Database) is a comprehensive classification system for membrane transport proteins, including ion channels. The annotation results of the genome of strain XJCJMh1 using the TCDB database show that the most abundant functional genes are related to primary active transporters, followed by electrochemical potential-driven transporters. This indicates that primary active transporters are the major membrane transport proteins in this strain. The results are presented in Figure 8F.

The predicted effectors of the strain include proteins involved in secretion systems and gene clusters related to secondary metabolism. Pathogens secrete these proteins into the extracellular environment or host cells through type N secretion systems (TNSS) to control immune responses and cell apoptosis, thereby causing pathological reactions. Among Gram-negative bacteria, the type III secretion system (T3SS) is commonly used to study pathogens at the molecular level, including infection mechanisms and virulence. In this strain, a total of 2644 annotated genes were predicted, of which 98 were type III secretion system effectors. Secondary metabolites are substances synthesized by microorganisms during a certain growth period, using primary metabolites as precursors. They have no clear function in microbial life activities and are not essential for growth and reproduction. This strain has three predicted secondary metabolic gene clusters, namely bacteriocin, leucine arylamidase (LAP), and β-lactam. Among them, leucine arylamidase has the most annotated genes, with a total of 52 genes, followed by β-lactam with 20 annotated genes. The results are shown in Figure 8G.

### 3.8. Virulence and Drug Resistance Analysis Results

The annotation results from the Pathogen-Host Interaction Database (PHI) are shown in Figure 8H. The results indicate that there are 239 genes where mutations lead to a reduction in pathogenicity of the pathogen, and 26 genes where mutations enhance pathogenicity. Additionally, mutations in 99 genes have no effect on pathogenicity. There are 21 genes where mutations result in a complete loss of pathogenicity. The database also identifies 4 lethal factors. Furthermore, there is 1 gene where a mutation confers resistance to chemicals, and another gene where a mutation increases sensitivity to chemicals.

The genome of strain XJCJMh1 was annotated with a total of 74 virulence genes in the VFDB (Virulence Factors Database). These genes were categorized based on their functions into several groups, including adhesion, biofilm formation, effector secretion systems, exoenzymes, exotoxins, immune modulation, motility, nutritional/metabolic factors, post-translational modification, regulation, stress survival, and others. The majority of the virulence factors were found in the categories of adhesion, immune modulation, and nutritional/metabolic factors, as shown in Table 3.

The annotation results from the CARD (Comprehensive Antibiotic Resistance Database) reveal that the whole-genome sequence of strain XJCJMh1 contains a variety of resistance genes. These include genes conferring resistance to tetracyclines, fosfomycin, fluoroquinolones, aminoglycosides, mupirocin, acetylphenazine antibiotics, β-lactams, lincosamides, diaminopyrimidines, aminocoumarins, sulfonamides, and glycopeptides. Additionally, the presence of multiple drug efflux pump genes was noted, which are key contributors to the multidrug resistance profile of this strain, as detailed in Table 4.

## 4. Discussion

Bovine Respiratory Disease Complex (BRDC) is a multifactorial disease affecting the global cattle industry, and its pathogenesis is closely related to the synergistic infection of *Mycoplasma bovis* (MB) and *Mannheimia haemolytica* (MH). Previous studies have confirmed that *Mycoplasma bovis* enhances the pathogenicity of *Mannheimia haemolytica* by disrupting the host’s respiratory mucosal barrier and immune defense system, and this synergistic effect is often a key factor leading to high mortality in calves [3]. This is consistent with the findings of Valeris-Chacin. *Mycoplasma bovis* creates an immunosuppressive environment for secondary infection by *Mannheimia haemolytica* by damaging the host’s respiratory mucosal barrier and immune defense system (such as impairing alveolar macrophage function and inhibiting lymphocyte proliferation), thereby significantly enhancing the pathogenic efficacy of *Mannheimia haemolytica* in BRDC. This synergistic effect ultimately exacerbates pulmonary inflammatory responses, induces sepsis, and other severe pathological changes, becoming a key pathogenic factor leading to calf death [6]. In this study, *Mannheimia haemolytica* strain XJCJMh1 was successfully isolated from the lung tissue of a 6-month-old calf infected with *Mycoplasma bovis*. The mouse pathogenicity experiment showed that this strain can cause pulmonary pathological damage and death, providing preliminary evidence for its potential role in the pathological process of BRDC.

The postmortem findings indicated that edema and congestion between myocardial fibers suggested myocardial inflammation; the presence of inflammatory cell infiltration and hepatocyte degeneration and necrosis in the liver indicated severe infection; disorganized splenic structure and necrosis indicated a severe impact on the immune system; inflammatory cell infiltration and vascular congestion in the lungs suggested that the lungs were the main site of infection; interstitial hemorrhage and renal tubular necrosis in the kidneys suggested that the kidneys were also affected by the infection, consistent with the systemic infection caused by *Mannheimia haemolytica* [26,27,28]. Studies have shown that mixed infections of *Mycoplasma bovis* and *Mannheimia haemolytica* can lead to more severe pathological changes [3]. Notably, the multi-organ lesions observed in our experiment share similarities with the pathological features reported in those co-infection studies. This observation raises the possibility that *Mannheimia haemolytica* alone may contribute to extensive tissue damage, and such multi-organ pathological features align with the findings from co-infection studies, suggesting this specific pathogen may have the potential to induce widespread tissue lesions that are comparable to those observed in co-infection scenarios.

In the mouse experiment, severe congestion and a small amount of hemorrhage in the lungs were similar to the pulmonary lesions in cattle, indicating that *Mannheimia haemolytica* can also cause severe pulmonary inflammation in mice. Severe hemorrhage and cellular necrosis in the mouse spleen indicated a severe impact on the immune system, consistent with the splenic lesions in cattle. Severe hemorrhage in the renal interstitium of mice suggested that the kidneys were also affected by the infection, possibly due to bacterial toxins or immune reactions. Notably, mice are not the natural host of *Mannheimia haemolytica*, and this experiment only observed pathological changes in mice without investigating the specific mechanisms underlying disease development.

In this study, we performed whole-genome sequencing of *Mannheimia haemolytica* strain XJCJMh1—isolated from the lung tissue of a diseased calf—using Illumina second-generation sequencing technology. Notably, Garzon et al. [29] compared WGS results (including virulence genes) of *Mannheimia haemolytica* isolates from diseased and healthy cattle and failed to identify a specific pathogenic pathotype. Consistent with this finding, the present study does not claim to define a unique pathogenic pathotype for XJCJMh1; instead, the WGS data of XJCJMh1 (deposited in GenBank) add to the existing body of genomic information on *Mannheimia haemolytica.*

GO database annotations revealed that XJCJMh1 harbors numerous genes enriched in cellular processes, metabolic processes, catalytic activity, binding, and cellular components. These genes are enriched in functional categories that are potentially involved in host adhesion, nutrient acquisition, and stress responses, which may contribute to the strain’s potential survival and adaptation in the host [30].

KEGG annotations further showed enrichment of genes in carbohydrate metabolism, amino acid metabolism, and cofactor/vitamin metabolism pathways, indicating robust metabolic and biosynthetic capacity. This suggests XJCJMh1 may have robust metabolic and biosynthetic capacity, which could potentially support its utilization of diverse nutrients for growth and reproduction—a trait that may be relevant to its potential colonization and infection of the host [31]. Consistently, COG functional classification showed genes primarily concentrated in translation systems and amino acid transport modules, reinforcing the strain’s evolutionary advantages in metabolism and biosynthesis from a protein function perspective.

Analyses using the PHI database and VFDB virulence gene library identified 26 virulence factors and 74 related coding genes in XJCJMh1, representing a set of factors that may be involved in multi-dimensional potential pathogenic processes. Type IV pili are associated with bacterial adhesion. Studies have shown that type IV pili play a role in DNA uptake, adhesion, and motility in Haemophilus influenzae, Pseudomonas aeruginosa, and Neisseria species, and they may perform similar functions in *Mannheimia haemolytica* [32,33]. *OmpA* may act as a ligand in *Mannheimia haemolytica*, participating in the binding of host cell receptor molecules, thereby playing a role in adhesion and colonization. Studies have shown that the surface-exposed loop regions of the *OmpA* protein vary among strains from different hosts (such as cattle and sheep), achieving precise regulation of tissue tropism, which may be related to host specificity [34].

The LuxS/AI-2 system operates as follows: at low cell density, the concentration of AI-2 is low, and the LuxS/AI-2 system receptor cannot detect a sufficient signal, leading to the activation of LuxO. This activates the transcription of Qrr1-4 sRNA, which inhibits the translation of HapR and promotes the expression of AphA. AphA activates virulence factors and biofilm formation. At high cell density, the concentration of AI-2 is high, and the receptor detects a sufficient signal, inactivating LuxO and inhibiting the transcription of Qrr1-4 sRNA. This leads to the expression of HapR, which inhibits the expression of AphA, thereby suppressing virulence factors and biofilm formation. This mechanism suggests that *Mannheimia haemolytica* may be more prone to initiating a new round of infections [35].

Lipopolysaccharide (LPS) is a component of the cell wall of Gram-negative bacteria and is the main endotoxin containing pathogen-associated molecular patterns (PAMPs) [36]. LPS activates macrophages via toll-like receptors (TLRs) and triggers the production of inflammatory cytokines, leading to sepsis [37]. LPS from *Mannheimia haemolytica* also induces an inflammatory cytokine response, leading to increased expression of β2-integrins in the host [38]. *Fur* is a transcriptional repressor that regulates the expression of iron uptake genes. *Mannheimia haemolytica* acquires iron through iron uptake proteins, thereby promoting its survival and reproduction within the host [39].

Additionally, iron uptake proteins may also affect bacterial pathogenicity by regulating the transcription and expression of leukotoxin (Lkt). Studies have shown that the availability of iron is crucial for the production of Lkt, which is one of the major virulence factors of *Mannheimia haemolytica* [40]. All the aforementioned virulence factors were detected in XJCJMh1, and these virulence factors may enhance its pathogenicity. The observed pulmonary pathology and mortality in XJCJMh1-infected mice, as well as organ damage in deceased calves, may be linked to the strain’s virulent gene expression. The animal pathogenicity experiments (alongside virulence gene detection) provide preliminary clues suggesting this strain may have pathogenic potential.

Comprehensive analysis using the CARD database revealed that *Mannheimia haemolytica* strain XJCJMh1 carries multiple resistance genes conferring resistance to 12 drug classes, including tetracyclines, fluoroquinolones, β-lactams, and sulfonamides. It also harbors drug efflux pump genes that actively expel antibiotics, reducing intracellular concentrations and diminishing bactericidal effects [41,42]. Co-expression of these efflux pumps may potentially induce cross-resistance, and this set of resistance-related genes suggests a potential complex resistance profile that could pose challenges for clinical treatment.

## 5. Conclusions

A *Mannheimia haemolytica* strain (XJCJMh1) was isolated from the lung tissue of a deceased *Mycoplasma bovis-infected* Simmental crossbred calf in Changji, Xinjiang. Pathogenicity experiments confirmed its pathogenicity, as XJCJMh1 infection caused mouse pulmonary pathology (hemorrhage, inflammatory exudation) consistent with bovine lung lesions. Genome data additionally identified that XJCJMh1 harbors multiple virulence and resistance genes that may confer enhanced pathogenic potential. This study, thus, demonstrates the presence of functional virulence genes and resistance determinants that may contribute to enhanced survival and host damage in co-infection contexts.

## Figures and Tables

**Table 1:** PCR Primers for *Mannheimia haemolytica* and Its Serotypes [16,17].

Primer Name	Primer Sequence (5′-3′)	Product Length (bp)
*UrvC*	F:TAATTTAGAAGCTTTAAATGAGCGC	238
	R:CATATCTAGGTCAATTAAGGCTTTG	
*gcp*	F:TGGGCAATACGAACTACTCGGG	227
	R:CTTTAATCGTATTCGCAG	
*Hyp*	F:CATTTCCTTAGGTTCAGC	306
	R:CAAGTCATCGTAATGCCT	
*Core2*	F:GGCATATCCTAAAGCCGT	106
	R:AGAATCCACTATTGGGCACC	
*TupA*	F:TGAGAATTTCGACAGCACT	78
	R:ACCTTGGCATATCGTACC	

**Table 2:** Basic Information on Genomic Components.

Components of the Genome	Number
Gene	2664
Tandem repeat, TR	
Tandem repeat sequence	85
Minisatellite DNA	64
Microsatellite DNA	8
Dispersed repetitive sequences	
LTR sequences	102
DNA transposons	35
Longinterspersed nuclear	36
elements, LINE	2
Short interspersed nuclear	5
elements, SINE	1
Rolling circle, RC	
Unknown	52
RNA ncRNA	6
tRNA	6
5S rRNA	2
16S rRNA	16
23S rRNA	3
sRNA	20
Genomics Islands, GIs	20
Prophage	
CRISPR	

**Table 3:** Annotation Results of Virulence Factors.

VF Category	Virulence Factors	Related Genes
Adherence	Type IV pili	pilQ
	Exopolysaccharide	mrsA/glmM, pgi
	Streptococcal enolase	Eno
	IlpA	IlpA
	OmpA	pomA
	HMW1/HMW2	HD_RS07760
	EF-Tu	tufA
Biofilm	AI-2	luxS
Effector delivery system	T6SS-II	clpV
Nutritional/Metabolic factor	Fur	Fur
	Iron/manganese transport	sitC, sitA
	HitABC	hitA, hitB
	HxuABC	hxuC, hxuB
	Achromobactin	DDA3937_RS07710
	Heme biosynthesis	hemE, hemA, hemN, hemC, hemY, hemL, hemB, hemM
Regulation	CsrA	csrA
	ClpP	clpP
Invasion	Capsular polysaccharide	rmlB, wecC, wbjD/wecB
	K1 capsule	neuB
	LPS	acpXL
	Capsule	bexA, bexB’, bexC’, bexD’, lipA, hcsB’, rpe
Stress survival	KatA	katA
Motility	Polar flagella	flmH
Exotoxin	Alpha-Hemolysin	hlyB, hlyD
Immune modulation	Hsp60	htpB Hsp60
	LOS	LpxC, kdsA, kdsB, opsX/rfaC, galU, gmhA/lpcA, orfM, lpsA, htrB, rfaE, waaQ, lgtF, lpxD, lpxA, galE, lpxK, msbB, lsgA, lsgD, lsgE, lsgF, kdkA, wecA, rfaD, manB/yhxB, kpsF, HD_RS06500, lpxH

**Table 4:** Annotation Results of Drug Resistance Genes.

ARO Category	ARO Name
Tetracyclines	tetQ, tet44, tet34
Fosfomycin	murA
Fluoroquinolones	gyrA, parE, mfd
Aminoglycosides	kdpE
Mupirocin	ileS
Acetanilidomycin	EF-TU
β-lactam	NmcR, PBP2, mecl
Lincosamides	clbA
Diaminopyrimidines	dfrD
Aminocoumarins	ParY
Sulfonamides	Sul2, leuO
Glycopeptides	VanTC, VanG, adeR, VanHO
Drug-resistant efflux pump	evgs, tetA(60), hmrM, CRP, mdtF, msrB, macA, oleC, tet(35), adeL, MexV, macB, lmrD, patA, tetA(60), rosB, qacH, tetA(48), cmeC, carA, sav1866, cpxA, rosA

## Data Availability

The original contributions presented in this study are included in the article/Supplementary Material. Further inquiries can be directed to the corresponding author.

## Supplementary Materials

The following supporting information can be downloaded at https://www.mdpi.com/article/10.3390/microorganisms13102258/s1. Figure S1: Isolation and PCR Identification Results of Bacteria from Lungs of Infected Mice; Figure S2: Detailed Diagrams of Lung Dissection and Pathological Sections from Infected Cattle; Table S1: Results of NR Database Functional Annotation for Genes in Genomic Islands.
