# Supplementary material for: Isolation, Pathogenicity and Genomic Analysis of Mannheimia haemolytica Strain XJCJMh1 in Bovine-Mycoplasma Co-Infection
Source: Microorganisms. 2025 Sep 26;13(10):2258. doi: 10.3390/microorganisms13102258 (PMC12566244; doi:10.3390/microorganisms13102258)
Supplement: Supplementary file 1 [file microorganisms-13-02258-s001.zip › FigS1.pdf]

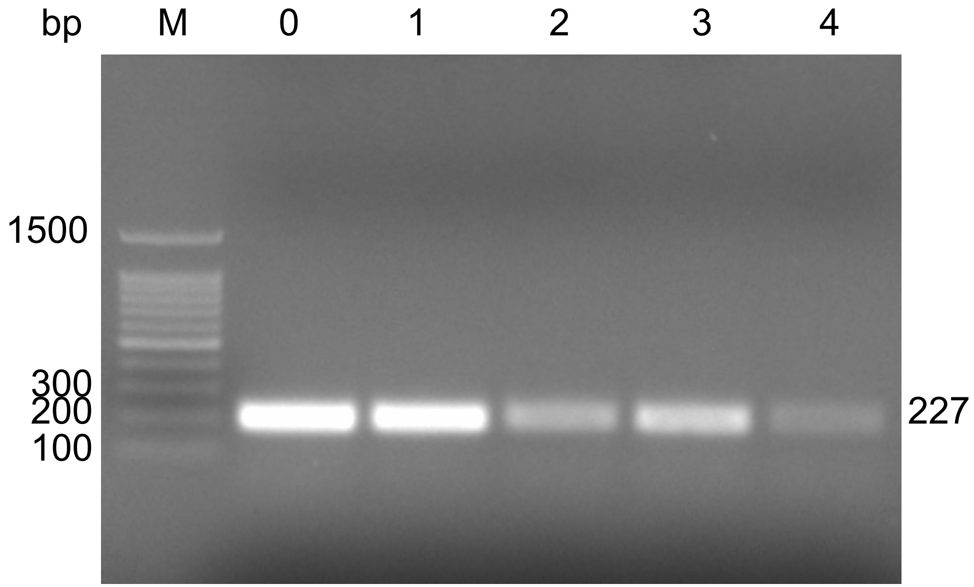

FigS1
Results of bacterial isolation and identification from the lungs of mice after challenge.
M: 100 bp DNA Ladder; 0: Positive control; 1, 2, 3, and 4: Bacterial detection of lung tissues from mice.
