# Supplementary material for: Isolation, Pathogenicity and Genomic Analysis of Mannheimia haemolytica Strain XJCJMh1 in Bovine-Mycoplasma Co-Infection
Source: Microorganisms. 2025 Sep 26;13(10):2258. doi: 10.3390/microorganisms13102258 (PMC12566244; doi:10.3390/microorganisms13102258)
Supplement: Supplementary file 1 [file microorganisms-13-02258-s001.zip › FigS2.pdf]

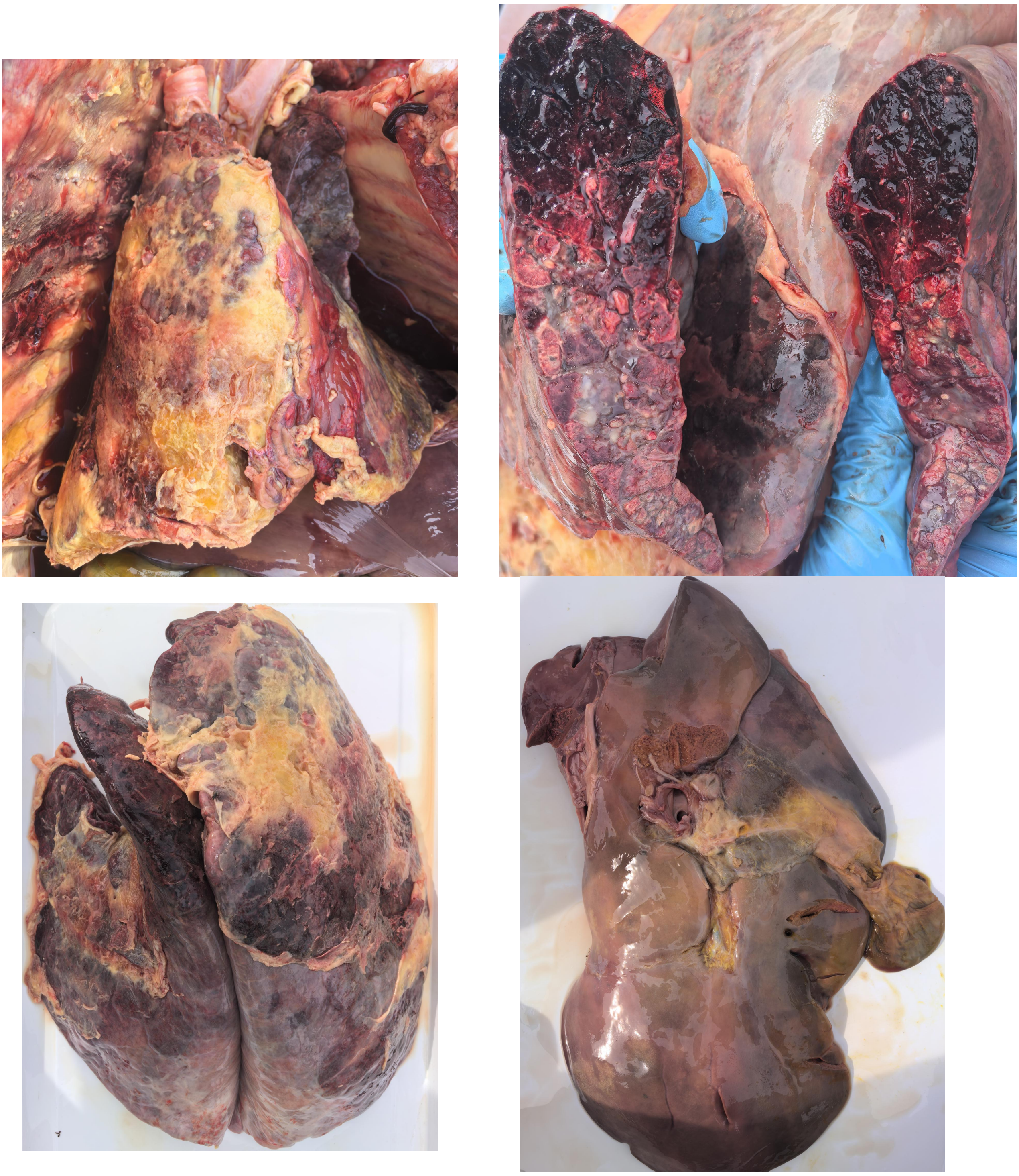

The above four images are the original pictures showing clearer pathological changes of some tissues and organs in Figure 2.

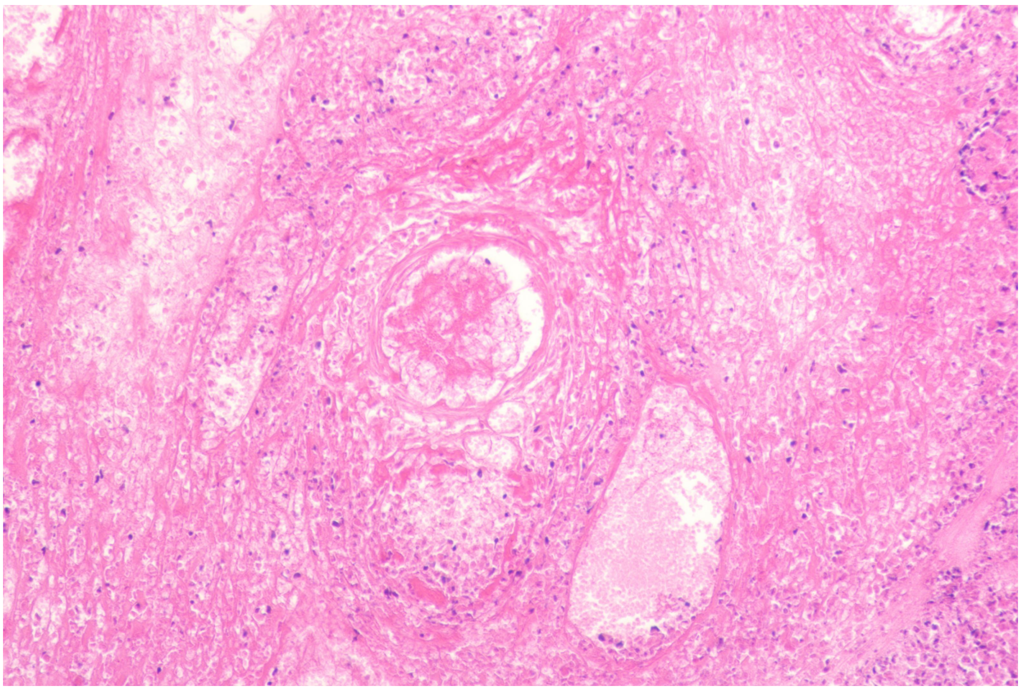

This section is a section of the lung tissue necrosis focus (200×), showing severe necrosis: karyolysis is observed in most cells, with only a small number of condensed cell nuclei (pyknosis) remaining; the normal tissue structure of the lung is severely damaged and difficult to identify.
