# Supplementary material for: Isolation, Pathogenicity and Genomic Analysis of Mannheimia haemolytica Strain XJCJMh1 in Bovine-Mycoplasma Co-Infection
Source: Microorganisms. 2025 Sep 26;13(10):2258. doi: 10.3390/microorganisms13102258 (PMC12566244; doi:10.3390/microorganisms13102258)
Supplement: Supplementary file 1 [file microorganisms-13-02258-s001.zip › TableS1.pdf]

Table 1: Results of NR Database Functional Annotation for Genes in Genomic Islands

GIs_ID	Gene_ID	Subject_ID	Subject_description
**GIs001**	gene0	WP_015484769.1	helix-turn-helix domain-containing protein [*Mannheimia haemolytica*]
	gene1	WP_015484768.1	hypothetical protein [*Mannheimia haemolytica*]
	gene2	WP_015484767.1	primase-like DNA-binding domain-containing protein [*Mannheimia haemolytica*]
	gene3	WP_209026252.1	Rha family transcriptional regulator [*Mannheimia haemolytica*]
	gene4	WP_006252014.1	AlpA family phage regulatory protein [*Mannheimia haemolytica*]
	gene5	WP_209026253.1	hypothetical protein [*Mannheimia haemolytica*]
	gene6	WP_006250286.1	hypothetical protein [*Mannheimia haemolytica*]
	gene7	WP_006250284.1	addiction module antidote protein [*Mannheimia haemolytica*]
	gene8	WP_238333094.1	type II toxin-antitoxin system RelE/ParE family toxin, partial [*Mannheimia haemolytica*]
	gene9	AGI31714.1	addiction module killer protein [*Mannheimia haemolytica* USDA-ARS-USMARC-183]
**GIs002**	gene0	WP_006250446.1	glutathione peroxidase [*Mannheimia haemolytica*]
	gene1	WP_006250447.1	DNA-binding transcriptional regulator OxyR [*Mannheimia haemolytica*]
	gene2	WP_006250448.1	HTH-type transcriptional repressor FabR [*Mannheimia haemolytica*]
	gene3	WP_006250449.1	DUF4298 domain-containing protein [*Mannheimia haemolytica*]
	gene4	WP_020828821.1	YadA-like family protein [*Mannheimia haemolytica*]
	gene5	WP_006250450.1	hypothetical protein [*Mannheimia haemolytica*]
	gene6	WP_147010826.1	adhesin [*Mannheimia haemolytica*]
	gene7	WP_006250451.1	ESPR domain-containing protein [*Mannheimia haemolytica*]
	gene8	EEY12532.1	putative cytochrome-c peroxidase [*Mannheimia haemolytica* serotype A2 str. BOVINE]
	gene9	WP_006250453.1	virulence RhuM family protein [*Mannheimia haemolytica*]
	gene0	WP_006250284.1	addiction module antidote protein [*Mannheimia haemolytica*]
	gene1	WP_238333094.1	type II toxin-antitoxin system RelE/ParE family toxin, partial [*Mannheimia haemolytica*]
	gene2	WP_006250240.1	hypothetical protein [*Mannheimia haemolytica*]
	gene3	WP_240025590.1	integrase arm-type DNA-binding domain-containing protein [*Mannheimia haemolytica*]
	gene4	WP_006253564.1	integrase arm-type DNA-binding domain-containing protein [*Mannheimia*

			haemolytica]
	gene5	WP_021280210.1	DUF927 domain-containing protein [*Mannheimia haemolytica*]
GIs003	gene6	WP_006250238.1	hypothetical protein [*Mannheimia haemolytica*]
	gene7	WP_006250237.1	hypothetical protein [*Mannheimia haemolytica*]
	gene8	WP_006250236.1	hypothetical protein [*Mannheimia haemolytica*]
	gene9	WP_006250235.1	ash family protein [*Mannheimia haemolytica*]
	gene1	AGQ24584.1	hypothetical protein F382_00550 [*Mannheimia haemolytica* D153]
	gene11	WP_006250233.1	hypothetical protein [*Mannheimia haemolytica*]
	gene12	ULX43875.1	helix-turn-helix domain-containing protein [*Mannheimia haemolytica*]
	gene13	WP_006250231.1	hypothetical protein [*Mannheimia haemolytica*]
	gene14	WP_006250230.1	hypothetical protein [*Mannheimia haemolytica*]
	gene15	WP_006250229.1	P2 family phage major capsid protein [*Mannheimia haemolytica*]
